# Molecular Identification of Cultured Microbial Population and Metabolic Impacts on Traditional Fermented *Motoho* From Different Regions of South Africa

**DOI:** 10.1155/ijfo/6621201

**Published:** 2026-09-16

**Authors:** Ngwekazi Nwabisa Mehlomakulu, Andries Masenge, Folarin Anthony Oguntoyinbo

**Affiliations:** ^1^ Department of Consumer and Food Sciences, University of Pretoria, Pretoria, South Africa, up.ac.za; ^2^ Department of Statistics, University of Pretoria, Pretoria, South Africa, up.ac.za; ^3^ A.R. Smith Department of Chemistry and Fermentation Sciences, Appalachian State University, Boone, North Carolina, USA, appstate.edu

## Abstract

Sorghum *motoho* is a fermented sorghum porridge produced through natural fermentation or backslopping. To understand the in situ microbial population and their metabolic roles during fermentation, water, *tomoso* (traditional starter culture), and *motoho* samples were collected from eight areas in the Free State and Eastern Cape provinces of South Africa. The highest lactic acid bacteria (LAB) population was 6.81 Log CFU/g in *motoho* (pH 3.97) from Area 3 and 6.78 Log CFU/g in *tomoso* (pH 4.43) from Area 6, whereas the lowest yeast population was 3.09 Log CFU/g in *tomoso* (pH 3.16) from Area 5. The highest population was 6.78 Log CFU/g in *motoho* (pH 3.93) from Area 8. Overall, the LAB population (5.74 Log CFU/g) was frequently isolated in *tomoso*, whereas the yeast population (5.36 Log CFU/g) was isolated in *motoho*. Selected LAB and yeast isolates were differentiated using RAPD‐PCR analysis using M13 primer; 15 and 23 distinct groups were observed among the 27 and 31 LAB and yeast isolates, respectively. Analysis of the 16S rDNA identified *Schleiferilactobacillus harbinensis*, *Levilactobacillus brevis*, *Lactiplantibacillus plantarum*, and *Limosilactobacillus fermentum* across seven areas, with *L. brevis* frequently isolated from *tomoso* samples of the six areas studied. Analysis of the D1D2 domain of the 26S rDNA identified the yeasts *Candida sake*, *Kazachstania unispora*, *Pichia kudriavzevii*, *Pichia fermentans*, and *Saccharomyces boulardii*, which the latter three were frequently isolated in *tomoso* and *motoho* from six areas. When compared with species from different regions and products, the *motoho* LAB isolates clustered together, whereas the yeast isolates clustered into eight groups on the neighbor‐joining phylogenetic tree. In the *tomoso* and *motoho* samples from all areas, the lactic acid concentration was detected between 0.04 and 1.97 mg/100 g, whereas the acetic concentration was below 0.36 mg/100 g. None of the LAB were amylolactic while the yeasts *Saccharomyces cerevisiae*, *C*. *sake*, and *S*. *boulardii* fermented maltose. The study revealed that the *tomoso* possessed a diverse microbial population, which consisted of different non‐*Saccharomyces* yeast species that survived the fermentation conditions.

## 1. Introduction

Sub‐Saharan Africa (SSA) countries such as South Africa rely on cereals as staple foods, mainly processed through spontaneous fermentation to produce thin or thick porridges and beverages at household level [1, 2]. Sorghum (*Sorghum bicolor* L. Moench) and maize (*Zea mays*) alone or in combination are often used to make these products. Spontaneous fermentation is employed due to its energy efficiency, long‐practiced use, and availability of starter culture in cases where fermentation through backslopping is applied.

Fermentation is an eon‐long food processing method, preceded by soaking cereal grains to soften the protein matrix of the grain, which allows for improved starch extraction [3, 4]. The activation of the endogenous enzymes—amylases and proteases—during this phase generates fermentable sugars, which facilitate the onset of fermentation by indigenous lactic acid bacteria (LAB). These LAB are reported to be amylolytic and possess *α*‐amylases that partially hydrolyze crude starch granules to monosaccharides or disaccharides [5]. Indigenous yeasts emerge when easily assimilable substrates are available and are reported to secrete hydrolytic enzymes; they assimilate the primary metabolites generated by endogenous enzymes or by LAB [6, 7]. This indigenous population plays a crucial role in the conversion of sorghum raw material to a finished product that is palatable, safe, and nutritious [8, 9].

Microbial and matrix interactions are the primary drivers of fermentation. The existence of both LAB and yeasts in fermented foods is based on direct and indirect microbial interactions such as mutualism, amensalism, commensalism, competition, or parasitism between yeast–yeast and/or yeast–bacteria species [10]. These interactions render beneficial effects to the fermented foods through the enrichment of the human diet and food substrates biologically with vitamins, protein, essential amino acids, and fatty acids; detoxification of food substrates; decreasing cooking time; increasing the shelf‐life of food; enhancing its flavor; and enriching food with probiotics [8, 11, 12].

Southern African sorghum fermented products include a thin porridge *motoho*, thick porridge *ting* and beer—*umqombothi* [2]. The LAB population in these products varies from *Lpb*. *plantarum*, *Limosilactobacillus fermentum*, *Lacticaseibacillus rhamnosus*, *Weissella cibaria*, *Lactococcus lactis*, *Enterococcus faecalis*, and *Lactobacillus paracasei* [3, 13–15], whereas the yeasts population identified in *motoho* were *Candida lambica*, *Candida kefyr*, *Candida glabrata*, *Candida pelliculosa*, *Rhodotorula mucilaginosa*, *Geotrichum candidum*, and *Geotrichum silvicola* [13]. Johansen et al. [10] reported on a total of 98 different yeast species from 43 indigenous SSA‐fermented foods. The yeast species include *S*. *cerevisiae*, *Pichia kudriavzevii*, *Candida tropicalis*, and *Kluyveromyces marxianus* were found in 77%, 60%, 47%, and 44% of the fermented products, respectively. *S*. *cerevisiae* is found as the frequently isolated fauna in indigenous sorghum‐based food products such *kisra*, *ogi*, *uji*, *koko*, *kenkey*, *injera*, and *mahewu* in West and East African countries [16].

With the rise of conscious consumers in Africa, there has been an increase in the market demand for functional foods that offer health benefits, convenience, meet the customer′s healthy lifestyle, and are still nutritionally adequate and safe. Thus, there is a need to look into indigenous and traditional foods to cater for this market. To achieve this goal, the microbial population responsible for the fermentation of *motoho* has to be properly characterized and their metabolic signature determined. Although there are preliminary studies that reported microbiota associated with *motoho* in South Africa [13, 17]. Up to date, the culturable microbial population of *motoho* and their corresponding metabolic impact is poorly understood.

The objective of this study was to use culture dependent and amplicon sequencing methods to determine the microbial diversity of artisanal‐produced *motoho* in different regions of South Africa and evaluate some of their fermentation metabolites. This is an important step for future development of starter cultures in the production of multifunctional fermented functional foods and beverages in Africa, as well as addressing food safety and nutrition challenges.

## 2. Materials and Methods

### 2.1. Sampling of *Motoho* in Different Regions of South Africa

**Figure 1:**
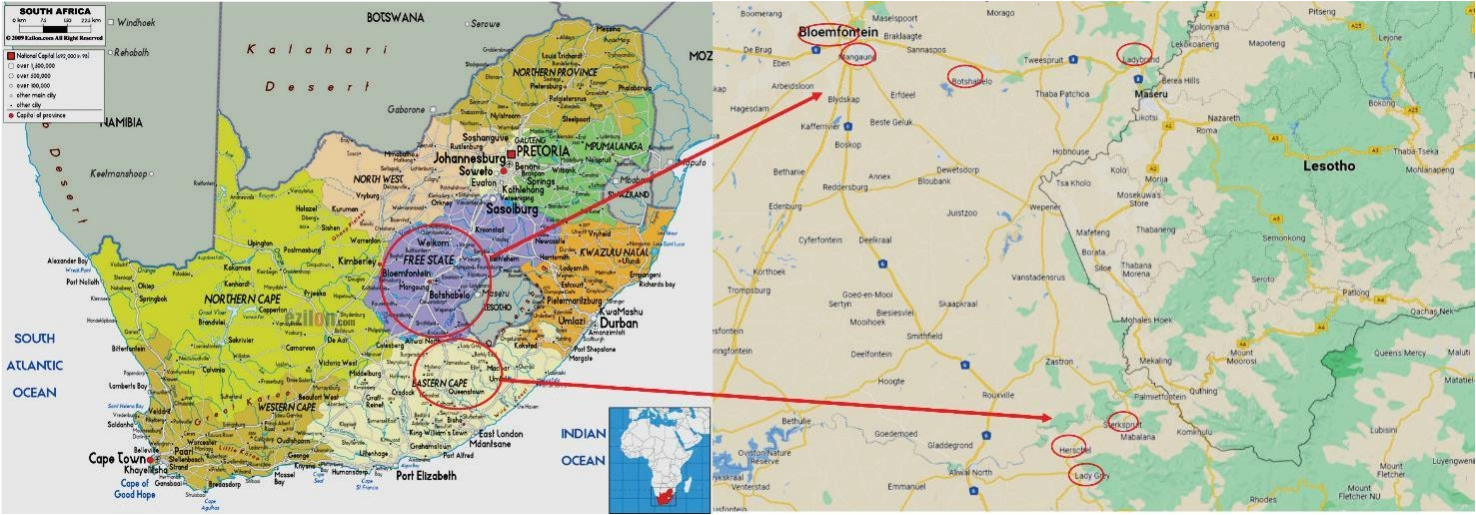
Collection sites of *motoho* samples in Free State and Eastern Cape provinces, South Africa.

Water (communal municipal tap water), *tomoso*, and *motoho* were collected from two South African provinces—Free State and Eastern Cape—during the period of September 19–23, 2023. In the Free State province, the sampled areas were as follows: Area 1 = Bloemfontein (Majakathatha), Area 2 = Bloemfontein (Central Park), Area 3 = Mangaung (Phase 2), Area 4 = Botshabelo, and Area 5 = Lady Brand. In the Eastern Cape province, the sampled areas were the following: Area 6 = Sterkspruit, Area 7 = Herschel, and Area 8 = Lady Grey (Figure 1). Duplicate samples were collected and immediately transferred from home containers (plastic bottles, glass jars, and pots) into washed and sterilized (with 70% ethanol) plastic bottles and the pH measured. All samples were kept in a cooler box with ice packs during transportation to the Department of Consumer and Food Sciences, University of Pretoria where they were stored at −80°C until analysis.

### 2.2. Microbial Population Determination

Thawed and cooled samples were processed by homogenizing 10 g or 10 mL of each sample in 90 mL maximum recovery diluent (Oxoid, United States) at 250 rpm for 30 s using a stomacher (Stomacher 400 Circulator, Seward, United States). The homogenate was 10‐fold serially diluted, and 100 *μ*L of respective dilutions were spread plated out on agar plates. Yeast peptone dextrose (YPD) agar 1% yeast extract (Alpha Biosciences, United States), 2% peptone (Fisher Scientific, United States), 2% dextrose (Alfa Aesar, United States), and 1.5% agar (Alfar Aesar, United States) supplemented with 100 mg/L chloramphenicol (Alfa Aesar, United States), pH 6.07 was used for the enumeration of yeasts. LAB were enumerated on De Man–Rogosa–Sharpe (MRS) agar (Alfa Biosciences, United States) supplemented with 0.1% cycloheximide (Acros Organics, Thermo Scientific, United States), whereas total aerobic counts were enumerated on nutrient agar (Criterion, Hardy Diagnostics, United States). The prepared YPD agar was sterilized in an autoclave (Yamato, Japan) at 121°C for 15 min at a pressure of 15 lbs per square inch. MRS and nutrient agar were prepared following the manufacturers′ instructions. YPD agar plates were incubated at 25°C for 3 days, whereas MRS and nutrient agar plates were incubated for 48 h at 37°C. Agar plates with CFU (colony forming units) between 30 and 300 were enumerated.

After the incubation period, colonies representative of yeasts and LAB based on phenotypic characteristics were subcultured on respective agar plates to obtain pure isolates. The obtained pure yeast and LAB isolates were aseptically inoculated, respectively, into YPD broth (pH 6.07), prepared with 1% yeast extract (Alpha Biosciences, United States), 2% peptone (Fisher Scientific, United States), 2% dextrose (Alfa Aesar, United States), or MRS (Alfa Biosciences, United States) broth, and incubated with shaking at 120 rpm for 18 h at 25°C and 37°C, respectively. YPD broth was sterilized in an autoclave as described above. Each isolate was cryopreserved in 50% (v/v) glycerol (Fisher Scientific, United States) and stored at −80°C.

### 2.3. Molecular Identification and Typing of Yeasts and LAB

#### 2.3.1. Whole Genome DNA Extraction

Eighteen‐hour axenic cultures of each yeast or LAB isolate were subjected to whole genome DNA extraction in an Eppendorf tube. The cells were harvested by centrifugation (Eppendorf 5430R, Germany) at 2000 × g for 10 min at 4°C. The pellet was resuspended with 1 mL of 10 mM Tris‐HCI (pH 8.5) (elution buffer) and centrifuged at 2000 × g for 2 min at 4°C, followed by washing with 400 *μ*L of the elution buffer at the same conditions and the supernatant discarded. The pellet was resuspended with 400 *μ*L of the elution buffer, and the cells were lysed with a mixture containing 20 *μ*L of 100 mg/mL lysozyme (Sigma, United States), 5 *μ*L of 10 U/*μ*L mutanolysin (Sigma, United States), 45 *μ*L of 20 mg/mL proteinase K (Sigma, United States), and 1 *μ*L of 10 mg/mL RNAse (Sigma, United States) and incubated at 37°C for 30 min. To the suspension, 70 *μ*L of 10% SDS (Avantor Performance Materials, LLC, United States) was added and mixed by inversion, followed by incubation at 65°C for 10 min. After incubation, 100 *μ*L of 5 M NaCI (Sigma, United States) was added and mixed, followed by 100 *μ*L of preheated CTAB (10% in 0.7 M NaCI) (Bioworld, United States). The suspension was vortex mixed until it turned white and incubated at 65°C for 10 min. To the mixture, 500 *μ*L of chloroform/isoamyl alcohol (24:1) (Alfa Aesar, United States and MP Biomedicals LLC, United States) was added and vortex mixed for 10 s, followed by centrifuging at 20 817 × g for 5 min at 4°C. The top clear layer was transferred to a clean Eppendorf tube and, to this, 300 *μ*L of ice‐cold isopropanol was added at a ratio of 0.5:500, followed by mixing and incubation at −20°C for 30 min. The mixture was centrifuged at 20 817 × g for 10 min at 4°C, and the supernatant was discarded. The pellet was washed with 500 *μ*L of cold 70% ethanol and centrifuged for 1 min under the same conditions. The supernatant was discarded, and the pellet was air dried for 5 min after which it was resuspended in 50 *μ*L of the elution buffer. To the suspension, 1 *μ*L of 10 mg/mL RNAse was added and incubated at 37°C for 30 min. To the mixture, 5 *μ*L of 3 M NaAcetate (pH 8.0) (Sigma, United States) and 100 *μ*L of ice‐cold 99% ethanol were added and mixed with gentle flicking. The mixture was centrifuged at 20 817 × g for 2 min, the supernatant was discarded, and the pellet was washed with 70% ice‐cold ethanol, followed by centrifuging under the same conditions. The supernatant was discarded, and the pellet was air dried for 5 min. The DNA was resuspended in 50 *μ*L of elution buffer, and the concentration was measured on a SpectraMax Quickdrop UV‐Vis Spectrophotometer (Molecular devices, United States).

#### 2.3.2. Random Amplified Polymorphic DNA

PCR was carried out on all the extracted DNA. The RAPD PCR reaction was prepared on a cooling block by mixing 20 *μ*L of the Taq Polymerase Master mix (Promega, United States), 5 *μ*L M13 primer (25 *μ*M) (Sigma, United States), 20 *μ*L nuclease‐free water (Promega, United States), and 5 ng/*μ*L of DNA template to a final volume of 50 *μ*L. Amplification was carried out on Biometra TOne thermocycler (Analytik Jena, United States) under the following conditions: initial denaturation at 94°C, 1 min followed by 35 cycles of denaturation at 94°C, 20 s; annealing at 40°C, 20 s; and extension at 72°C, 2 min with a final extension at 72°C for 2 min. A total of 5 *μ*L of each amplicon was visualized by running on a 2% ultrapure agarose gel (Invitrogen, United States) at 90 V for 1 h 30 min with a 1‐kb DNA ladder (Thermo Scientific, United States) after staining with ethidium bromide (Sigma, United States) for 1 h. The banding pattern for all the yeast and LAB amplicons was, respectively, compared with each other and grouped based on their banding pattern. From each yeast or LAB group, only one isolate was selected for amplification of the 16S rDNA and 26S rDNA D1D2 region of bacteria and yeasts, respectively.

#### 2.3.3. Identification of Yeast and LAB by Sequencing of the rDNA

Amplification of the 16S rDNA region of the LAB amplicons was prepared by mixing the PCR reaction on a cooling block with the following reagents: 20 *μ*L of Taq Polymerase Master mix, 2 *μ*L of 20 *μ*M 08F primer (5 ^′^‐AGAGTTTGATCCTGGCTCAG‐3 ^′^) and 2 *μ*L of 20 *μ*M 1391R primer (5 ^′^‐GACGGGCGGTGTGTRCA‐3 ^′^) (Sigma), 24 *μ*L nuclease‐free water and 5 ng/*μ*L of DNA template to a final volume of 50 *μ*L. Amplification was carried out on a Biometra TOne thermocycler under the following conditions: initial denaturation at 95°C, 3 min followed by 35 cycles of denaturation at 95°C, 30 s; annealing at 53°C, 30 s and extension at 72°C, 1 min 30 s, and final extension at 72°C, 10 min. The D1D2 of the 26S rDNA region of the yeast isolate amplicons was amplified using the reaction mixture: 20 *μ*L of Taq Polymerase Master mix, 2 *μ*L of 20 *μ*M NL1 primer (5 ^′^‐GCATATCAATAAGCGGGGAAAAG‐3 ^′^) and 2 *μ*L of 20 *μ*M NL4 primer (5 ^′^‐GGTCCGTGTTTCAAGACGG‐3 ^′^) (Sigma), 24 *μ*L nuclease‐free water and 5 ng/*μ*L of DNA template to a final volume of 50 *μ*L. Amplification was carried out on a Biometra TOne thermocycler under the following conditions: initial denaturation at 96°C, 5 min followed by 25 cycles of denaturation at 96°C, 10 s; annealing at 60°C, 1 min, and extension at 72°C, 2 min and final extension at 72°C, 2 min. A total of 5 *μ*L of each amplicon from the PCRs was visualized by running on a 2% ultrapure agarose gel at 90 V for 1 h 30 min with a 1‐kb DNA ladder followed by staining with ethidium bromide for 1 h.

#### 2.3.4. Gene Sequencing Identification

The 16S rDNA and D1D2 amplicons were purified with SureClean Plus (Meridian Life Science Inc., United States) following the manufactures′ instructions and the DNA concentration measured with SpectraMax Quickdrop UV‐Vis Spectrophotometer. Each of the purified amplicons were prepared for sequencing. For yeast identification, 10 *μ*M of the NL1 primer and 5 ng/*μ*L of the purified amplicon DNA were sent for sequencing of the D1D2 region at Eurofins Genomics LLC, United States. While for the LAB, 10 *μ*M of each 08F and 1391R primer were separately mixed with 5 ng/*μ*L of the purified amplicon DNA and sent for sequencing of the 16S rDNA region at Eurofins Genomics LLC. The obtained D1D2 sequences were analyzed with Finch TV software (Geospiza, United States). The 16S rDNA sequences were analyzed with Finch TV and Bioedit (Informer Technologies Inc., United States) to align the sequences. All obtained sequences identified using Basic Local Alignment Search Tool on the NCBI website (https://blast.ncbi.nlm.nih.gov/Blast.cgi?PROGRAM=blastn%26PAGE_TYPE=BlastSearch%26LINK_LOC=blasthome). The identified yeast and LAB isolates were clustered and compared with each other using the Molecular Evolutionary Genetics Analysis (MEGA II) software [18]. From the aligned sequences, neighbor‐joining trees were constructed for the LAB and yeast isolates.

#### 2.3.5. Carbon Substrate Fermentation and Starch Hydrolysis

Identified yeast isolates were tested for their ability to ferment the sugars—glucose, maltose, and fructose. Broth media consisting of 1% yeast extract, 2% peptone, and 1% of each of the sugars—dextrose (Alfa Aesar, United States), maltose (Fisher Scientific, United States), and fructose (Acros Organics)—were prepared with distilled water. To each media preparation, 0.02% of bromocresol purple was added. A total of 10 mL of each medium were dispensed into test tubes, and a Durham tube was inserted in these tubes prior to sterilization at 121°C for 15 min. Once cooled, 100 *μ*L of each yeast isolate biomass was inoculated into each test tube, and these were incubated for 48 h at 25°C with shaking at 120 rpm. Prior to inoculation, the yeast isolates were cultured by inoculating a single pure colony of each isolate into 10‐mL YPD broth prepared as described above and incubating overnight at 25°C with shaking at 120 rpm. Biomass of the grown cultures was harvested by centrifuging at 3000 rpm for 5 min at room temperature. The supernatant was discarded, and the pellet was washed with sterile distilled water under the same conditions. The supernatant was discarded and the pellet resuspended in 500 *μ*L sterile distilled water, and this was used to inoculate the prepared media. The experiment was repeated in triplicate.

The hydrolysis of starch was tested in the LAB isolates. Starch agar medium was prepared by dissolving 0.3% beef extract, 1% soluble starch, and 1.2% agar. The media was sterilized at 121°C for 15 min and poured into sterile petri dishes once cooled to 60°C. To each agar plate 10 *μ*L of each LAB isolate was spotted and allowed to dry at room temperature. The agar plates were incubated at 37°C for 48 h. After incubation, each agar plate was flooded with iodine solution to observe a zone of clearance around the spotted colony. The LAB isolates were prepared by culturing a single pure colony in 10‐mL MRS broth at 37°C overnight without shaking. Biomass of the grown cultures was harvested by centrifuging at 3000 rpm for 5 min at room temperature. The supernatant was discarded, and the pellet was washed with sterile distilled water under the same conditions. The supernatant was discarded and the pellet resuspended in 100 *μ*L sterile distilled water, and this was used to spot the prepared agar plates. The experiment was repeated in triplicate.

#### 2.3.6. Determination of Fermentation Metabolites

Fermentation metabolites—glucose, maltose, fructose, lactic, and acetic acid—concentration in the collected samples was determined from 10 g of each of the samples that were homogenized by vortex mixing, followed by centrifuging at 2750 × g for 10 min at 4°C. A total of 600 *μ*L of cell‐free supernatants were deproteined with 50 *μ*L of Carrez A and 50 *μ*L of Carrez B reagent (Megazyme, United States). To this mixture, 100 *μ*L of 0.1 M NaOH was added, and the mixture was diluted with ultrapure water to a final volume of 1 mL. The mixture was centrifuged at 14,500 × g for 5 min at 4°C. To the collected supernatant, 50 mg of basic and acid resins Amberlite IR 120(OH) and IR 45 (I) (Laboratory Reagents, Philadelphia, Pennsylvania, United States) were added then mixed for 10 min and left to stand at room temperature for 5 min. After centrifugation at 14,500 × g for 5 min at 4°C, the supernatants were diluted fivefold and filtered with 0.2‐*μ*m syringe filters (Sigma, United States). All the metabolites were analyzed by HPLC using the Aminex HPX‐87H Column, 300 × 7.8 mm (Biorad, United States) operated at oven temperature of 35°C, flow rate of 1.5 mL/min. Distilled water was used as the mobile phase for the sugar analysis, whereas 5 mM H_2_SO_4_ was used the mobile phase for organic acid analysis. All standards were prepared by dissolving with distilled water and filtered with 0.2‐*μ*m syringe filters. The sugar standards (Acros Organics, United States) range was 0.02, 0.015, 0.01, 0.005, and 0.002 g/mL, whereas the organic acids (Thermo Scientific and Sigma, United States) range was 10, 5, 2.5, 1.25, 0.5, 0.2, 0.1, 0.05, and 0.025 g/L.

### 2.4. Statistical Analysis

Descriptive statistics, means and standard deviation were performed on the water, *tomoso*, and motoho samples using Microsoft Excel Office 365. Due to the small sample size, the nonparametric Kruskal–Wallis *H* test was used to examine whether the distribution of the microbial population (*H* statistic) differed significantly across sampling areas for the water, *tomoso*, and *motoho* samples. When significant differences were detected, post hoc pairwise comparisons with Bonferroni‐adjusted *p* values were performed. The same procedure was applied to investigate differences in the distributions of lactic acid and acetic acid concentrations across sampling areas for both *tomoso* and *motoho* samples. Analyses were conducted using IBM SPSS Statistics (Version 30, 2024), and statistical significance was established at *α* = 0.05.

## 3. Results

### 3.1. pH of Samples

Pure water samples collected exhibited a neutral pH range between 7 and 8.3. *Tomoso* samples were prepared through backslopping, which were fermented for a period of a week, as reported by the producer. The *tomoso* pH ranged from 3.16 to 4.43, whereas the *motoho* pH ranged from 3.26 to 4.23 (Table 1).

**Table 1:** pH measurement of water, sorghum *tomoso*, and *motoho.*

Area	Cereal	Water	*Tomoso*	*Motoho*
1	Sorghum + maize	3.53* ± 0.00	3.50 ± 0.00	3.93 ± 0.00
2	Sorghum + maize	np	3.22 ± 0.00	3.26 ± 0.00
3	Sorghum	7.67 ± 0.00	3.54 ± 0.00	3.97 ± 0.00
4	Sorghum	7.26 ± 0.00	3.74 ± 0.00	4.03 ± 0.00
5	Sorghum	8.03 ± 0.00	3.16 ± 0.00	3.76 ± 0.00
6	Sorghum + maize	8.26 ± 0.00	4.43 ± 0.00	4.23 ± 0.00
7	Sorghum	7.12 ± 0.00	3.43 ± 0.00	3.68 ± 0.00
8	Sorghum	7.65 ± 0.00	3.53 ± 0.00	3.93 ± 0.00

*Note:* Asterisk “*” denotes liquid/water from *tomoso* prepared from backslopping, which was used to cook *motoho*.

Abbreviations: np, not provided; water, communal tap water.

The Kruskal–Wallis test revealed statistically significant differences across areas for all samples. Specifically, the results indicated significant variation in pH for water *H*(6) = 13.00, *p* = 0.043; *tomoso* *H*(7) = 15.00, *p* = 0.036; and *motoho* *H*(7) = 15.00, *p* = 0.036 (Table 1). These findings suggest that the pH measurements were not uniformly distributed across the sampled areas for any of the collected samples. To further explore these effects, pairwise comparisons were performed. Several unadjusted comparisons reached significance (e.g., water: Areas 1–6, *p* = 0.004; *tomoso*: Areas 5–6, *p* = 0.003; and *motoho*: Areas 2–6, *p* = 0.003). However, after applying the Bonferroni correction for multiple testing, none of the pairwise comparisons remained significant (all adjusted *p* > 0.05). This indicates that, although the omnibus Kruskal–Wallis tests identified significant area effects, specific area‐to‐area differences could not be confirmed with high statistical confidence once the adjustment for multiple comparisons was applied. These results likely reflect the influence of the small sample size.

### 3.2. Characterization and Identification of Microbial Isolates

Total aerobic counts of the water, *tomoso*, and *motoho* samples collected in all areas were between 3.07 and 7.03 Log CFU/mL or g. In the water samples, aerobic population was below limit of detection for Areas 1, 2, 4, 5, and 8; whereas the same was observed for the *tomoso* samples from Areas 4 and 7. The yeast population ranged from 3.09 to 6.44 Log CFU/mL and 4.15 to 6.78 Log CFU/g for the *tomoso* and *motoho* samples, respectively, in the eight areas sampled. In all areas, the yeast population in the water samples was below the limit of detection. Area 6 *tomoso* had the highest yeast population at 6.44 Log CFU/mL, whereas *motoho* samples from Area 8 had the highest population at 6.78 Log CFU/g.

The LAB population of *tomoso* samples in Areas 5 and 7 was below the limit of detection, whereas Area 5 *motoho* samples had the lowest LAB population at 3.52 Log CFU/g. A population greater than 6.00 Log CFU/mL was observed for the *tomoso* samples from Areas 1, 2, 3, and 6 (Table 2). Only *motoho* samples from Areas 1, 3, and 7 had a similar LAB population. No LAB population was detected in the water samples from all areas. Phenotypically, LAB were identified as gram‐positive, catalase‐negative rod‐coccoid. Acetic acid bacteria as gram‐negative catalase positive and yeast were first grouped as apiculate, elongated, and spherical from a microscopic analysis (data not shown).

**Table 2:** Microbial population in water, sorghum *tomoso*, and *motoho*.

Area	TAC			Yeasts		LAB	
	Water (Log CFU/mL)	*Tomoso* (Log CFU/mL)	*Motoho* (Log CFU/g)	*Tomoso* (Log CFU/mL)	*Motoho* (Log CFU/g)	*Tomoso* (Log CFU/mL)	*Motoho* (Log CFU/g)
Area 1	nd	4.55 ± 0.03	5.92 ± 0.60	4.90 ± 0.51	6.07 ± 0.16	6.62 ± 0.11	6.04 ± 0.22
Area 2	nd	7.03 ± 0.16	3.96 ± 0.59	4.66 ± 0.22	4.94 ± 0.10	6.73 ± 0.51	4.90 ± 0.12
Area 3	3.13 ± 0.29	6.08 ± 2.40	6.50 ± 0.36	5.44 ± 0.56	4.23 ± 1.13	6.22 ± 2.29	6.81 ± 0.07
Area 4	nd	nd	4.62 ± 0.19	nd	4.25 ± 0.45	4.08 ± 0.44	4.44 ± 0.16
Area 5	nd	3.38 ± 0.09	4.31 ± 0.22	3.09 ± 0.01	4.15 ± 0.21	nd	3.52 ± 0.52
Area 6	3.07 ± 0.44	6.62 ± 0.07	3.57 ± 0.30	6.44 ± 0.11	5.92 ± 0.18	6.78 ± 0.00	3.60 ± 0.18
Area 7	5.01 ± 0.17	nd	6.45 ± 0.05	nd	6.54 ± 0.01	nd	6.49 ± 0.03
Area 8	nd	5.40 ± 1.46	3.65 ± 0.07	4.98 ± 1.18	6.78 ± 0.00	4.02 ± 0.68	4.74 ± 1.36
	3.74	5.51	4.87	4.92	5.36	5.74	5.07

Abbreviation: nd, below limit of detection (30–300 CFUs).

Kruskal–Wallis tests were performed for microbial population to assess total aerobic counts, yeasts, and LAB differences in the areas within each treatment. No statistically significant differences were observed in the areas for any of the microbial groups. The total aerobic count in the water treatment samples did not differ significantly in the areas (*p* = 0.223). The *tomoso* total aerobic counts, yeasts, and LAB were all nonsignificant (*p* = 0.285, 0.211, and 0.172, respectively) in the areas. Similarly, for motoho, tests for total aerobic counts, yeasts, and LAB yielded *p* values of 0.061, 0.064, and 0.056, respectively, in the areas.

### 3.3. Identification of Yeast and Bacteria Isolates by Sequencing of the 26S and 16S rDNA

**Figure 2:**
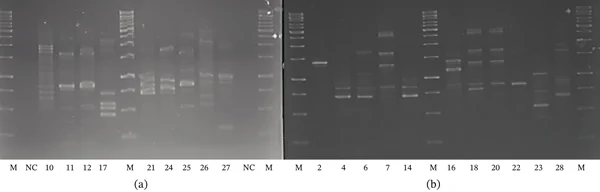
(a,b) *Motoho* yeast isolate RAPD‐PCR.

**Figure 3:**
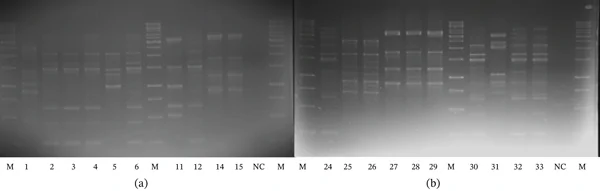
(a,b) *Motoho* LAB isolate RAPD‐PCR.

Based on the total microbial population determined above, 31 yeast and 25 LAB isolates were identified. Isolates with similar phenotypic characteristics were grouped and selected for RAPD‐PCR. The analysis of M13 RAPD PCR identified 23 and 15 groups of yeast (Figure 2a,b) and LAB (Figure 3a,b) isolates, respectively, based on band pattern. To be able to identify respective members of the RAPD‐PCR group with a similar banding pattern, one isolate was selected for D1D2 or 16S rDNA PCR sequencing for yeast and LAB identification, respectively.

**Figure 4:**
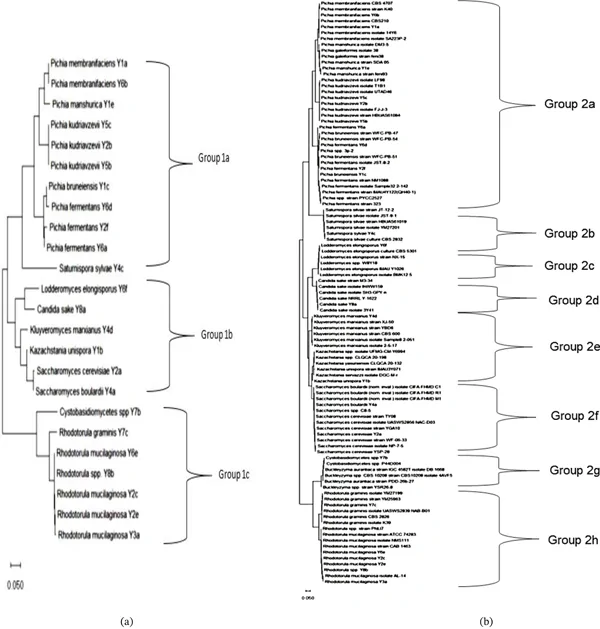
(a) Dendrogram showing multiple sequence alignment of D1D2 gene sequences of yeasts isolated from *motoho*. (b) Dendrogram showing comparison of yeast species isolated from *motoho* with yeast species from different samples and regions.

Sequence analysis of the yeast isolates on the NCBI nucleotide database yielded 16 frequently identified species from all the samples and these were 99%–100% similar to the species listed in Tables 3, S1, S2, and S3. *Pichia bruneiensis*, *P*. *kudriavzevii*, *Pichia fermentans*, *S*. *boulardii*, and *R. mucilaginosa* were overall the frequently identified species. In the *tomoso* and product samples, *Pichia*, *Saccharomyces* species, and *R. mucilaginosa* were frequently isolated, respectively (Table 3). All the identified yeast species were found to be genetically different from each other based on the neighbor‐joining phylogenetic tree, and three major genera clusters were identified: *Pichia* and *Saturnispora =* 1a; *Candida*, *Kluyveromyces* and *Saccharomyces* = 1b, and *Rhodotorula* = 1c (Figure 4a). The D1D2 sequences of the identified species were compared with species with which they share 99%–100% similarity on the NCBI database and were then grouped based on genera (Figure 4b). The *motoho Pichia* isolates clustered (Group 2a) with *Pichia* species from different sources and regions, whereas the *Saturnispora* isolate was clustered separately (Group 2b). Species in Group 1b (Figure 4a) were further separated, with *Candida* and *Lodderomyces* isolates differentiated into new clusters (Groups 2c and 2d), respectively. *Kluyveromyces* and *Kazachstania* clustered into (Group 2e). All the *Saccharomyces* species clustered into (Group 2f). The species in Group 1c (Figure 3a) separated into two clusters, *Cystobasidiomycetes* (Group 2 g) and *Rhodotorula* (Group 2 h) (Figure 4b).

**Table 3:** Yeast species identified in water, sorghum *tomoso*, and *motoho*.

Species identified	Water	*Tomoso*	*Motoho*	Area
*Pichia membranifaciens*		2		1 and 6
*Kazachstania unispora*		1		1
*Pichia bruneiensis*		2	1	1, 2, and 4
*Pichia manshurica*		1		1
*Saccharomyces cerevisiae*		2		2 and 6
*Pichia kudriavzevii*		2	2	2 and 5
*Rhodotorula mucilaginosa*			4	2, 3, and 6
*Pichia fermentans*		2	2	2 and 6
*Saccharomyces boulardii*		2	1	1, 4, and 5
*Saturnispora silvae*			1	4
*Kluyveromyces marxianus*			1	4
*Candida sake*		1		8
*Rhodotorula graminis*	1			7
*Lodderomyces elongisporus*			1	6
*Cytobasidiomycetes*	1			7
*Rhodotorula* spp.			1	8
Total yeasts identified	2	15	14	

**Figure 5:**
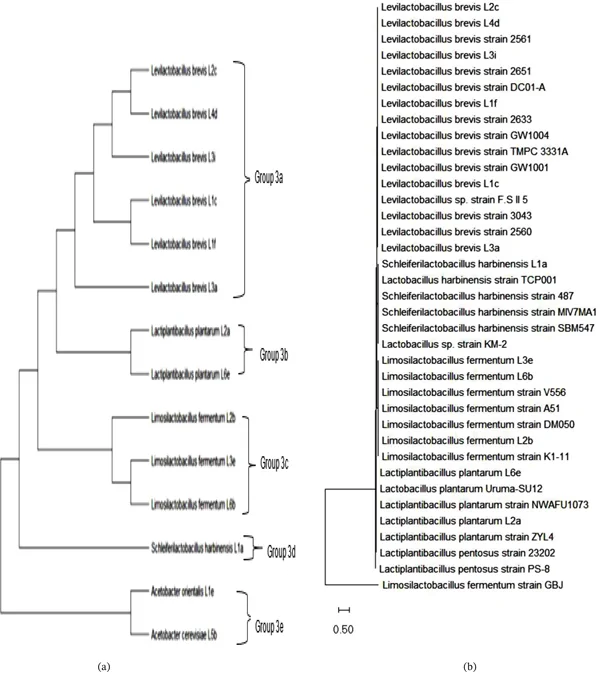
(a) Dendrogram showing multiple sequence alignment of 16S rRNA gene sequences of bacteria isolated from *motoho*. (b) Dendrogram showing comparison of bacteria species isolated from *motoho* with sequences of bacteria isolated from different samples and regions.

Sequence analysis of the isolated LAB on the NCBI nucleotide database yielded four frequently isolated species that were distributed in the *motoho* samples and these were 99%–100% similar to the species listed in Table 4. *Levilactobacillus brevis* was the frequently isolated species followed by *Lb. fermentum* in the *motoho* samples. In *motoho* from Areas 1 and 5, the species *Acetobacter cerevisiae* (two isolates) and *Acetobacter orientalis* (one isolate) were identified. Clustering of the *motoho* LAB and *Acetobacter* isolates exhibited four clades of the LAB species identified (Groups 3a, b, c, and d), whereas the *Acetobacter* isolates clustered together—Group 3e (Figure 5a). Comparison of the isolates with species that share > 99% similarity revealed two clades, separating the genera *Levilactobacillus*, *Schleiferilactobacillus*, and *Limosilactobacillus* from *Lactiplantibacillus* (Figure 5b).

**Table 4:** LAB species identified in water, sorghum *tomoso*, and *motoho*.

Species identified	Water	*Tomoso*	*Motoho*	Area
*Schleiferilactobacillus harbinensis*	1			1
*Levilactobacillus brevis*		9	7	1, 2, 3, 4, 6, and 7
*Lactiplantibacillus plantarum*		2		2 and 6
*Limosilactobacillus fermentum*		4	2	2, 3, and 6
Total LAB identified	1	15	9	

### 3.4. Determination of Fermentation Metabolites

In all the collected samples, none of the sugars—glucose, fructose, and maltose—were detected. Area 1 exhibited negligible concentration of 0.01 mg/100 g of glucose and fructose. The *tomoso* of Areas 1 and 3 exhibited higher lactic acid concentration (1.03 and 1.97 mg/100 g, respectively) compared with the *tomoso* from the other areas (Table 5). Area 6 *tomoso* had no detectable lactic acid. With the *motoho* products, the lowest lactic acid concentration was recorded for Area 6 at 0.04 mg/100 g, whereas the highest concentration for the *motoho* samples—0.58 mg/100 g, which was detected in Area 8. Acetic acid was only detected in *tomoso* samples from Areas 1, 3, 4, 5, and 7. No acetic acid was detected in the rest of the areas as well as the *motoho* samples from all sampled areas (Table 5). Lactic acid concentration in the *tomoso* and *motoho* revealed no significant differences across areas (*p* = 0.125 and *p* = 0.070, respectively). Similarly, acetic acid concentration had no significant differences across areas (*tomoso* *p* = 0.120).

**Table 5:** Lactic and acetic acid concentration in sorghum *tomoso* and *motoho*.

Areas	Lactic acid (mg/100 g)		Acetic acid (mg/100 g)	
	*Tomoso*	*Motoho*	*Tomoso*	*Motoho*
Area 1	1.03 ± 0.09	0.11 ± 0.03	0.04 ± 0.03	—
Area 2	0.55 ± 0.05	0.16 ± 0.04	—	—
Area 3	1.97 ± 0.25	0.16 ± 0.00	0.04 ± 0.01	—
Area 4	0.56 ± 0.18	0.08 ± 0.01	0.08 ± 0.06	—
Area 5	0.55 ± 0.03	0.11 ± 0.04	0.36 ± 0.01	—
Area 6	—	0.04 ± 0.02	—	—
Area 7	0.21 ± 0.01	0.05 ± 0.00	0.18 ± 0.01	—
Area 8	0.55 ± 0.49	0.58 ± 0.59	—	—

*Note:* Em dash “—“ denotes not detected.

### 3.5. Carbon Substrate Fermentation and Starch Hydrolysis

The identified yeast species were tested for their ability to ferment the sugars—glucose, fructose, and maltose. All the yeasts fermented glucose and fructose. Only the yeasts *S. cerevisiae*, *C. sake*, and *S. boulardii* fermented maltose. However, none of the fermented sugars led to gas production, whereas none of the identified LAB and yeast species hydrolyzed starch.

## 4. Discussion

In each of the South African provinces, sample collection sites were based on popular areas, where sorghum *motoho* was sold and consumed by commuters and households. *Motoho* is produced for traditional ceremonies and the production follows the traditional method as described previously [2]. In the Free State province, three households were sampled, whereas in the Eastern Cape, one household was sampled. In both provinces, water used for food preparation is tap water supplied by the municipality of the area and was sampled to trace the source of the microorganisms throughout the making of the product.

The pH of all the water samples was neutral, with the exception of Areas 5 and 6. This is attributed to the different water treatments in the different production areas. The acidic pH between 3.43 and 4.43 in the *tomoso* is attributed to an active fermentative state of the samples. Similar pH ranges were observed in the *motoho* samples correlated to other fermented sorghum products previously studied [14, 19]. The reduced pH is due to the organic acids produced during fermentation. Lactic acid fermentation through heterofermentative pathway is mainly characteristic of sorghum fermentations with lactic acid and acetic acid as the most detected organic acids in nonalcoholic beverages, foods, and alcoholic beverages [20–22]. In the *motoho* samples, lactic acid concentration of 1.03 and 1.97 mg/100 g was detected in Areas 1 and 3 *tomoso* samples. In both samples, the heterofermentative *Lb. brevis* was identified in the *tomoso* and *motoho*. In all areas, the cooked product (*motoho*) had less than 0.16 mg/100 g of lactic acid except for Area 8, where the organic acid was not different from the *tomoso*. In our data, the secondary organic acid, acetic acid was only detected in the *tomoso* samples but not in the *motoho* samples. During fermentation, the LAB are responsible for acidification of the matrix through the production of lactic acid. From our microbial analysis, the heterofermentative LAB population was between 4 and 7 Log CFU/g in the *tomoso* sample—the active fermentative state. Even though heat was applied in the cooking of the *tomoso* to make *motoho*, a quantifiable LAB population was observed in *motoho*, which contributed to the organic acid concentration. Tested sugars in *tomoso* were undetectable due to rapid consumption of the sugars by the LAB for production of lactic acid. In artisanal production, the fermentation of *tomoso* is completed within 12–48 h as determined by the sour taste [2]. All collected samples were of artisanal production; thus, the observed population is attributed to the resident microbial population in the environment, preparation, and cooking utensils.

The identified LAB (Figure 3a,b) and yeast (Figure 2a,b) isolates could be differentiated by RAPD‐PCR using M13 primer. This primer has been reported to differentiate LAB [23–25]. The differentiating power of RAPD‐PCR has been reported in discriminating yeasts and bacteria from mixed microbial ecosystems [26–31]. Clustering of the LAB and yeast isolates from the eight areas clearly exhibited shared species in the *tomoso* and *motoho* from different areas. In all areas, the *tomoso* proved to be species‐rich and diverse compared with *motoho*. In particular, the species associated with sorghum fermentation were also identified in this current study. This is attributed to the methods used. Amplification of the 16S rDNA and D1D2 regions has been proven to be a robust and reproducible method to identify species from diverse ecological sites of LAB and yeasts [32, 33].

Greppi et al. [19] reported on *C. krusei*, *Cl. lusitaniae*, and *S. cerevisiae* in *ogi*, with *C. krusei* as the frequently isolated yeast. *Gowé*, a nonalcoholic sorghum beverage was dominated by the yeasts *C. krusei*, *C. tropicalis*, and *K. marxianus* [34], whereas *P. kudriavzevii* and *K. marxianus* were frequently isolated species in spontaneous fermented *mawé* at a population range of 80%–100% and 60%–80%, respectively [35]. As observed in the current study, *P. kudriavzevii* was isolated in *tomoso* and *motoho* samples from Areas 2 and 5, representing the Free State province. Similarly, the yeasts *P. bruneiensis*, *P. fermentans*, and *S. boulardii* were also isolated from two or more areas in the provinces studied. Non‐*Saccharomyces* are prevalent in African‐fermented foods and beverages with *S. cerevisiae* often isolated from alcoholic beverages [10]. In the water samples from Area 1 in the Free State province, only *Rhodotorula graminis*, *Cytobasidiomycetes*, and the LAB *Schleiferilactobacillus harbinensis* species were identified. Moodley et al. [13] identified *R. mucilaginosa* in laboratory‐prepared motoho, whereas in this study, this species was identified in the products of three areas—2, 3, and 6—found in the Free State and Eastern Cape, respectively.

*Lb. brevis* was the most frequent LAB in both the *tomoso* and *motoho*. The species was isolated in all areas except for Area 8. This indicates the wide distribution of the species in the production of sorghum *motoho*. This is in agreement with the household samples of *bushera* as studied by Muyanja et al. [21]. *Lb. fermentum* was the second frequently identified species in three areas in the current study. This species is reported to also be frequently isolated in *gowé* fermentation [34]. Notably, in the laboratory prepared *motoho, Lb. fermentum*, and *Lpb. plantarum* were identified, with the former being the most frequent species [13]. In *gowé*, *Lb. fermentum* was the most frequent in motoho [34]. In this study, *Lpb. plantarum* was only identified in *tomoso* in Areas 2 and 7, which represented the Free State and Eastern Cape province, respectively. It is generally agreed that LAB are prevalent in traditional fermented cereal beverages in Africa [9, 36–40]. Similar fermented sorghum products also exhibited varied LAB species distribution as observed by [41–43]. In the current study, *Lpb. plantarum* was not frequently isolated and this could be attributed to regional differences. A similar result was reported during *kunu-zaki* fermentation in West Africa [38]. The LAB species identified in the study were found to lack amylolactic activity, which can be attributed to strains differences.

Both LAB and yeasts contribute to functional, sensory, nutritional, health, and physicochemical properties of fermented products due to the interactions with the matrix and diverse species during fermentation [10]. Based on these favorable attributes, several studies have attempted to use yeasts and LAB in coculture as starters for the production of fermented sorghum beverages [20, 44, 45].

The yeast *P. kudriavzevii* has been reported to have probiotic properties, intracellular folate production, and possesses the phytase enzyme [46–48]. In an earlier study, *P. kudriavzevii* strain OG32 was investigated for the production of a functional food based on these key characteristics. Fermentation with the strain exhibited survival in the cereal throughout fermentation, radical scavenging activity, and enhanced flavor and viscosity of the product [48]. Furthermore, strains of *P. kudriavzevii* were found to increase the essential amino acids phenylalanine, lysine, valine, and leucine with a total free amino acid increase of 47%, 20%–45%, and 79.4% at 24, 48, and 72 h, respectively, in fermented *mawé* dough [48]. Whereas *S. boulardii* has been reported and is commercially used as a probiotic yeast [49, 50]. In the current study, the *P. kudriavzevii* and *S. boulardii* isolates hold potential for use in the production of functional fermented foods based on their ability to survive the processing conditions as cocultures with LAB. This is the first report of isolation of *S. boulardii* in an African‐fermented cereal food. This study deliberately identified the cultured microbes in *motoho* with the aim of using them as multifunctional starter cultures during industrial production. Future studies on the biodiversity of microbial population in *motoho* using culture‐independent techniques and a wider sampling area are recommended for a more accurate representation of microbial species within the *motoho* ecosystem, which may pose safety concern or possess desirable functional properties. On the overall, it is established that some identified LAB and yeast were frequently isolated across different regions of South Africa where *motoho* is produced. Candidate strains of LAB could be used for the production of *motoho*; however, their other functional advantages should be further studied.

## Funding

This study was supported by the National Research Foundation, South Africa (129767); Fulbright South African Research Scholar Program; and Appalachian State University (10.13039/100009708; URC Grant # 2341).

## Ethics Statement

The project received ethical clearance NAS011/2021 issued by the University of Pretoria, South Africa.

## Conflicts of Interest

The authors declare no conflicts of interest.

## Supporting information


**Supporting Information** Additional supporting information can be found online in the Supporting Information section. Supporting data are included as Tables S1, S2, and S3.

## Data Availability

The data that support the findings of this study are available from the corresponding author upon reasonable request.
