# Supplementary material for: Molecular Identification of Cultured Microbial Population and Metabolic Impacts on Traditional Fermented Motoho From Different Regions of South Africa
Source: Int J Food Sci. 2026 Sep 16;2026:6621201. doi: 10.1155/ijfo/6621201 (PMC13583440; doi:10.1155/ijfo/6621201)
Supplement: Supplementary file 1 [file IJFO-2026-6621201-s001.docx]

**Supplementary Data**

List of tables

Table S1: Assigned accession numbers for the yeast isolates identified in sorghum

Table S2: Assigned accession numbers for the lactic acid bacteria isolates identified in sorghum

Table S3: Assigned accession numbers for the acetic acid bacteria isolates identified in sorghum

Table S1:

| **Yeast species** | **Isolate number** | **Assigned NCBI accession number** |
| --- | --- | --- |
| *Pichia membranifaciens* | Y1a | PZ577228 |
| *Kazachstania unispora* | Y1b | PZ577229 |
| *Pichia bruneiensis* | Y1c | PZ577230 |
| *Pichia manshurica* | Y1e | PZ577231 |
| *Saccharomyces cerevisiae* | Y2a | PZ577232 |
| *Pichia kudriavzevii* | Y2b | PZ577233 |
| *Rhodotorula* *mucilaginosa* | Y2c | PZ577234 |
| *Rhodotorula* *mucilaginosa* | Y2e | PZ577235 |
| *Pichia fermentans* | Y2f | PZ577236 |
| *Rhodotorula mucilaginosa* | Y3a | PZ577237 |
| *Saccharomyces boulardii* | Y4a | PZ577238 |
| *Saturnispora silvae* | Y4c | PZ577239 |
| *Kluyveromyces marxianus* | Y4d | PZ577240 |
| *Pichia kudriavzevii* | Y5b | PZ577241 |
| *Pichia kudriavzevii* | Y5c | PZ577242 |
| *Pichia fermentans* | Y6a | PZ577243 |
| *Pichia membranifaciens* | Y6b | PZ577244 |
| *Pichia fermentans* | Y6d | PZ577245 |
| *Rhodotorula mucilaginosa* | Y6e | PZ577246 |
| *Lodderomyces elongisporus* | Y6f | PZ577247 |
| *Cystobasidiomycetes* spp. | Y7b | PZ577248 |
| *Rhodotorula graminis* | Y7c | PZ577249 |
| *Candida sake* | Y8a | PZ577250 |
| *Rhodotorula spp.* | Y8b | PZ577251 |

Table S2:

| **Lactic acid bacteria** | **Isolate number** | **Assigned NCBI accession number** |
| --- | --- | --- |
| *Schleiferilactobacillus harbinensis* | L1a | PZ567958 |
| *Levilactobacillus brevis* | L1c | PZ567959 |
| *Levilactobacillus brevis* | L1f | PZ567960 |
| *Lactiplantibacillus plantarum* | L2a | PZ567961 |
| *Limosilactobacillus fermentum* | L2b | PZ567962 |
| *Levilactobacillus brevis* | L2c | PZ567963 |
| *Levilactobacillus brevis* | L3a | PZ567964 |
| *Limosilactobacillus fermentum* | L3e | PZ567965 |
| *Levilactobacillus brevis* | L3i | PZ567966 |
| *Levilactobacillus brevis* | L4d | PZ567967 |
| *Limosilactobacillus fermentum* | L6b | PZ567968 |
| *Lactiplantibacillus plantarum* | L6e | PZ567969 |

Table S3:

| **Acetic acid bacteria** | **Isolate number** | **Assigned NCBI accession number** |
| --- | --- | --- |
| *Acetobacter orientalis* | L1e | PZ568047 |
| *Acetobacter cerevisiae* | L5b | PZ568048 |
